# Optimizing gamma radiation shielding with cobalt-titania hybrid nanomaterials

**DOI:** 10.1038/s41598-023-33864-y

**Published:** 2023-06-01

**Authors:** Islam G. Alhindawy, M. I. Sayyed, Aljawhara H. Almuqrin, Karem A. Mahmoud

**Affiliations:** 1 Nuclear Materials Authority, P.O. Box 530, El-Maadi, Cairo, Egypt; 2 Department of Physics, Faculty of Science, Isra University, Amman, Jordan; 3 Department of Nuclear Medicine Research, Institute for Research and Medical Consultations (IRMC), Imam Abdulrahman Bin Faisal University (IAU), P.O. Box 1982, 31441 Dammam, Saudi Arabia; 4 Department of Physics, College of Science, Princess Nourah Bint Abdulrahman University, P.O.Box 84428, 11671 Riyadh, Saudi Arabia; 5 Ural Federal University, 19 Mira St, Yekaterinburg, Russia 620002

**Keywords:** Chemical engineering, Materials chemistry, Physical chemistry

## Abstract

Cobalt-doped titania nanocomposites were fabricated to be utilized for radiation shielding aims. The chemical composition of the composites was measured using the energy-dispersive X-ray spectrometer. Moreover, the structure of the composites was evaluated using the X-ray diffractometer, and the morphology of the fabricated composites was presented using the scanning electron microscope. Furthermore, the γ-ray shielding properties were estimated using the Monte Carlo simulation between 0.059 and 2.506 MeV. The linear attenuation coefficient of the fabricated composites decreased by factors of 93% for all samples by raising the incident γ-energy between 0.059 and 2.506 MeV. Moreover, the partial replacement of the Ti^4+^ by Co^3+^ slightly enhanced the linear attenuation coefficient from 0.607 to 0.630 cm^−1^ when the Co^3+^ increased from 0 to 3.7 wt%. The improvement in the linear attenuation coefficient causes an enhancement in other radiation shielding properties.

## Introduction

High-energy ionizing radiations such as X-rays are used in applications such as radiation therapy to eliminate cancerous cells and for imaging of the body. This form of electromagnetic radiation is also used in the field of energy production, agriculture, and many others, with new uses for this technology created every year^1–3^. Because ionizing radiation has such high energy, these photons can also induce negative side effects on the human body such as acute radiation poisoning, cancer, and death. A common technique to reduce these effects is to use a radiation shield that absorbs incoming photons and lowers the amount of radiation to a safe level^4–6^. Nanomaterials outperform conventional radiation shields due to their small particle size, which results in more even dispersion of the filler heavy metal oxides (HMOs) that are introduced into the shield. Greater dispersion means that the incoming radiation has a higher probability to get absorbed or deflected by the atoms within the material, leading to greater attenuation. Nanoparticles are becoming widely used in the construction of cement building materials such as cement paste, mortars, and concretes, which enhance the properties of the materials when introduced with nano HMOs^7–12^.

Titanium oxide nanostructures are often used as an antimicrobial agent for the food packaging industry or as photocatalysts for the degradation of organic compounds because these nanostructures are simple to process, have a low cost, and their ability to induce these reactions can easily be tuned without sacrificing the thermal or chemical stability of the material. Nano-TiO_2_ is chemically stable, which allows it to be used in cement materials as a filler to improve the chemical characteristics of the material. Ti also has a greater total neutron reaction cross section than Ca and Si at most energy regions, which are used to make conventional cement. This characteristic leads to cement paste containing nano-TiO_2_ having a better neutron shielding capability than plain cement paste^13–17^.

Additionally, TiO_2_ itself has a low cost, can abundantly be found, is not toxic, and is chemically inert. It has extensively been used in the coating industry, in waste-water purification, and energy storage devices. TiO_2_ as a typical n-type semiconductor has a carrier concentration of only 10^17^–10^18^ cm^−3^ and has a high refractive index at visible wavelengths. Pure TiO_2_ has three different polymorphs, each with its bandgap energy. More specifically, these are rutile (for 3.0 eV), anatase (for 3.2 eV), and brookite (for ~ 3.2 eV). The bandgap of TiO_2_ can be tuned by doping it with various ions or defects, which activates the TiO_2_ compound in the visible light spectrum; pure TiO_2_ is active in the ultraviolet region. By introducing transition metal oxides and noble metal compounds into TiO_2_, visible light TiO_2_ photocatalysts are created, which can be used to remove water pollutants. Cobalt oxides are one of these dopants that are drawing attention because of their god speed in the photocatalytic-reduction of Carbon dioxide (CO_2_) in visible and ultravilot light and dye-sensitized solar cells for energy production^18,19^.

To understand the shielding abilities of certain materials, radiation shielding parameters need to be calculated and analyzed across a wide range of energies. Monte Carlo simulations are used to determine these values such as linear attenuation coefficients using a specific set of conditions. These simulations can be used to understand the properties of material before experimentally determining these parameters, to save time and resources, or alongside theoretical calculations such as XCOM to ensure the two methods agree with each other. After the initial values are obtained, further parameters can be determined to gain a full understanding of the radiation shielding abilities of the material^20–25^.

The concept presented in present study is estsblished on the exact planning of a two dimintion (2D) nanoparticular structure of cobalt oxides and titanium oxides linked by carbon sheets. The inclusion of cobalt contributes to thermal stability, corrosion resistance, and wear, which makes it, along with titanium, useful in many industries. Additionally, cobalt oxides stand out due to their capacity for coloring. The two-dimensional carbon sheets increase the surface area of the prepared nanomaterial. The newly synthesized compounds' characterization and γ-ray protection particulares has been inspected.

## Methods and materials

### Manufacture of materials

(I) A known size and concentration (0.4 mL–0.1 M) of hydrous potassium chloride, 98 mL solution of ethanol, and 1.6 mL of (Ti_4_O_28_H_12_C) titanium isopropoxide have been mixed together and agitated for 5 h. All the contents were then moved to a Teflon-lined stainless steel autoclave with a 100 mL capacity.Then the temperature was raised to 170 °C for 35 h. After that, the autoclave has been brought to cooling naturally at room temperature. The finished product was gathered thoroughly and soaked in ethanol and has been dried in the oven at 70 °C after filtering. Finally, the solids were transferred to the furnace to be calcined at a temperature of 500 °C for 4 h to produce TiO_2_ NPs. (II) to produce a Cobalt-doped TiO_2_ nanocomposite (Co-TiO_2_), 0.9 gm of cobalt acetate is added to the mixed solution before transferring it to the autoclave and then following the same previous steps. (III) The method for preparing cobalt-doped TiO_2_ loaded on carbon sheets (Co-TiO_2_/C) involves mixing 0.5 g of Co-TiO_2_ nanocomposites produced in step (ii) with 80 mL of an aqueous solution containing 0.02 g glucose. The mixture has been stirred for 4 h, then placed in the autoclave used in the previous steps then heated for 30 h at 170 °C. After that, leave the autoclave to cool naturally in the air. The solids were gathered thoroughly and stirred well in water for washing and have been arid in the furnace at 70 °C. Finally, the solids powder have been calcined for 4 h at 500 °C.

### Characterization of the fabricated materials

Utilizing a (PANalytical X'Pert Pro diffractometer, Netherlands), XRD patterns have been obtained. To comprehend the crystallographic phase, a monochromatic CuKa radiation source with a wavelength of (0.15406 nm) was set to (45 kV) and (40 mA) at 25 °C. The diffractograms were captured with a step size of (0.013) over a scan range of 20°–80°. To predestine the morphology of the fabricated solid powders materials and appreciation the element analysis, scanning electron (SEM) microscope and an energy-dispersive X-ray spectrometer (EDX) havebeen used (JEOL JSM-6400—Jeol Ltd., Tokyo, Japan). The TEM analysis, which was performed using a (Philips CM200, America) transmission electron microscope with a 200 kV accelerating voltage, provided information about the morphological structure of the composite. The crystallinity of various components in the produced composite was determined using Raman spectroscopy with a 532 nm laser excitation (GL Gem Raman™ PL532, Canada).

### γ-ray shielding capacity estimation

**Figure 1:**
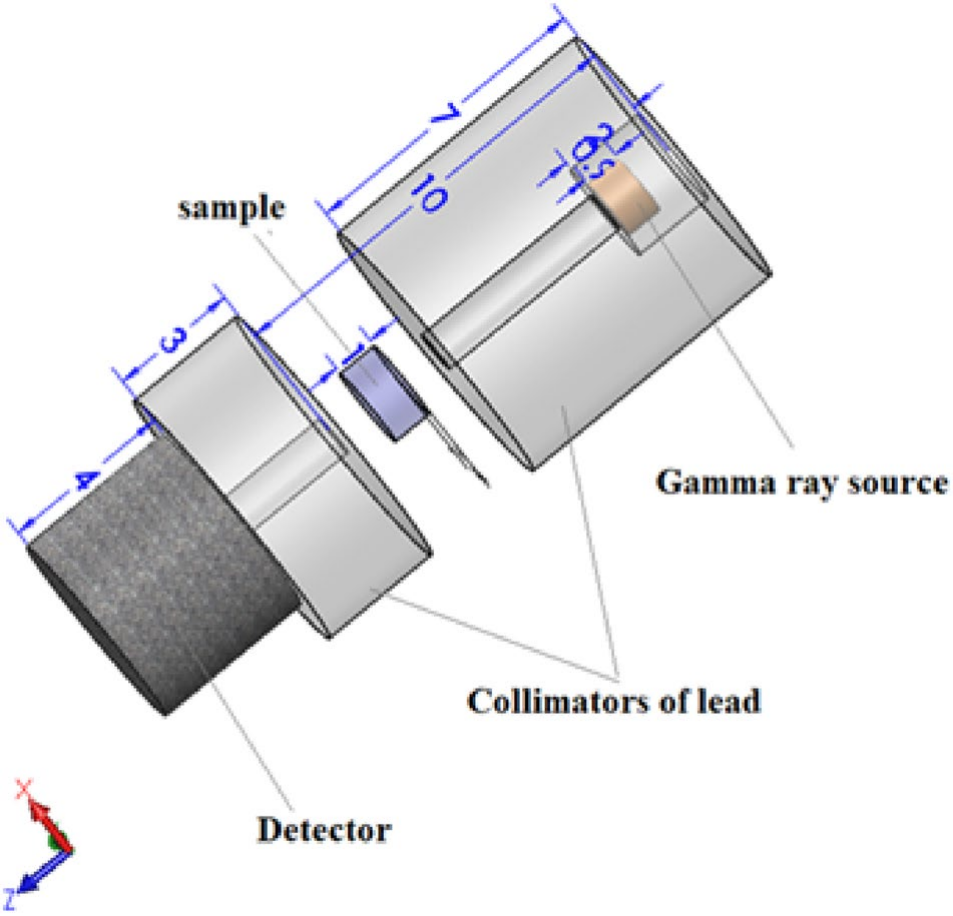
The MCNP’s geometry as illustrated in the input file.

Utilizing the MCNP5 code, the ability for γ-ray shielding for the newly synthesized cobalt-doped titania nanocomposites was simulated in the γ-ray energy ambit of (0.059–2.506 MeV). The MCNP code utilized the nuclear database ENDF/B.VI.8 and the tally F4 to estimate the median flux per unit cell and average track length (ATL) of gamma photons in the chosen composites. To estimate the ATL of gamma photons in the manufactured materials an input file contains all information about the fabricated nanocomposites, radioactive source, detector, and tally, as illustrated in Fig. 1. The required information was introduced to the MCNP’s input file through some definite cards such as cell, surface, materials, source, and tally cards. After runout the simulation process, a new output file was created and has a txt format. The created output file contains all required information about the ATL, the number of collisions in the material cell as well as the relative error in the simulation process. The relative error recorded for all samples ranged from ± 3%. Detailed information about created geometry and the dimensions of the geometry components collimators, sample, detector, and source was discussed in some previous works^26,27^.

Using some mathematical equations, the simulated MCNP’s ATL for the fabricated composites was transferred to the linear (µ) and mass (µ_m_, cm^2^/g) attenuation coefficients. Then, based on the estimated µ, the half-value thickness (Δ_0.5_), lead equivalent thickness (Δ_eq_), transmission factor (TF), and radiation protection efficiency (RPE) were evaluated as illustrated in the following equations^28^.

1 $$\mu \left( {{\text{cm}}^{ - 1} } \right) = \frac{{\text{I}}}{{\text{x}}}\ln \left( {\frac{{N_{o} }}{{N_{t} }}} \right)$$

$N_{0}$, $N_{t}$, and x are the number of emitted photons from the radioactive source, the transmitted photon number through the fabricated composite with a definite thickness (x, cm).

2 $$\mu_{m} = \frac{{\mu \left( {{\text{cm}}^{ - 1} } \right)}}{{\rho \left( {\frac{g}{{{\text{cm}}^{3} }}} \right)}}$$

The fabricated composite’s thickness that can attenuate 50% of the emitted γ- photons define the Δ_0.5_ and it is can be predicted through Eq. (3).

3 $$\Delta_{0.5} \left( {{\text{cm}}} \right) = \frac{\ln \left( 2 \right)}{\mu }$$

4 $$\Delta_{eq} \left( {{\text{cm}}} \right) = \frac{{\left( {\ln \left( {N_{o} } \right) - \ln \left( {N_{t} } \right)_{pb} } \right) \times x_{composite} }}{{(\ln \left( {N_{0} } \right) - \ln \left( {N_{t} } \right)_{composite} )}}$$

5 $$TF \left( \% \right) = \frac{{N_{t} }}{{N_{o} }} \times 100$$

6 $$RPE \left( \% \right) = \frac{{\left( {N_{o} - N_{t} } \right)}}{{N_{o} }} \times 100$$

## Findings and discussion

### Description of the fabricated composites

#### X-Ray diffraction analysis analysis (XRD)

**Figure 2:**
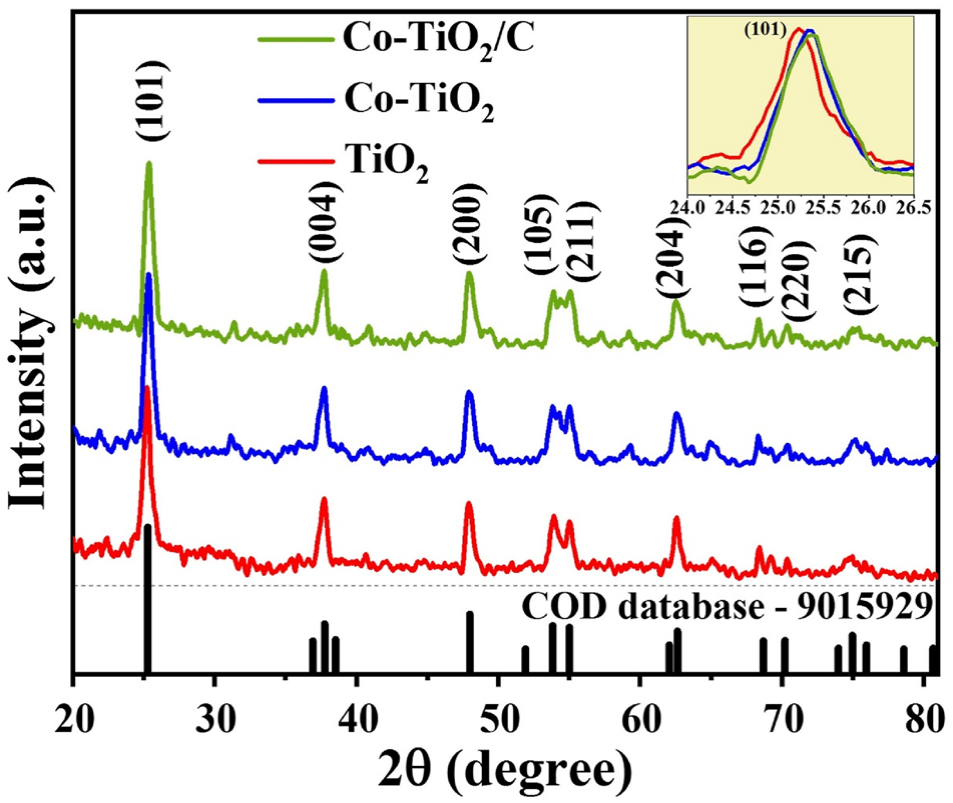
XRD diffraction pattern for the fabricated nanocomposites samples.

**Figure 3:**
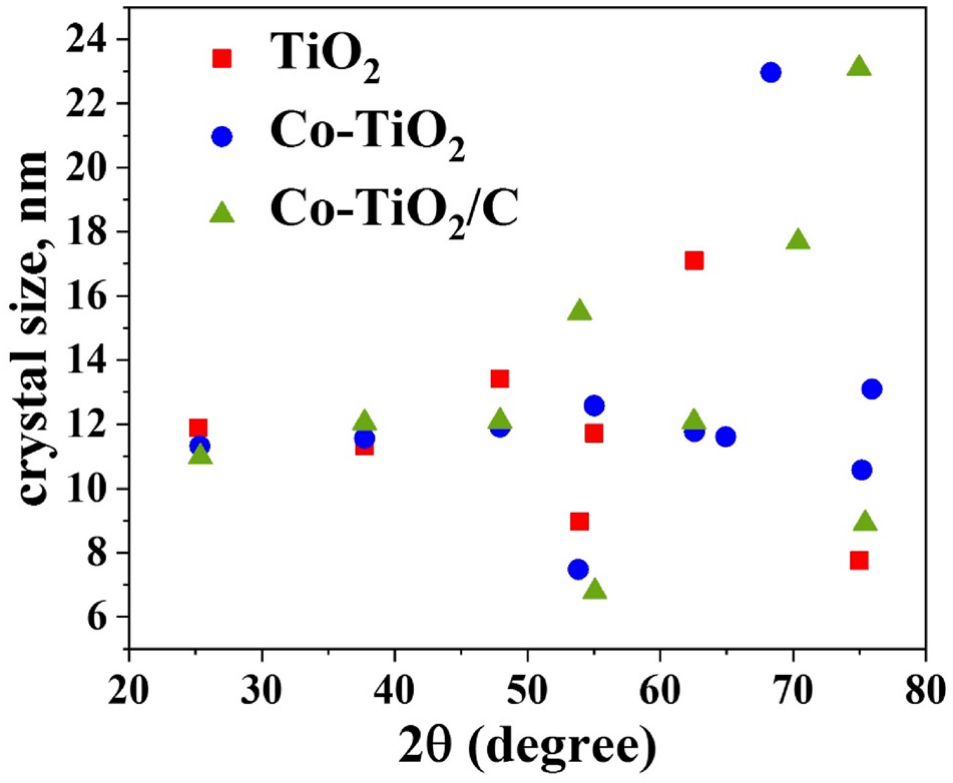
The crystal size of the fabricated nanocomposites samples.

The XRD diffraction pattern (Fig. 2) was utilized to illustrate the synthesized composites’ phase compositions. TiO_2_ XRD pattern shows all of the tetragonal anatase characteristic peaks (JCPDS 9015929) which the main diffraction peaks of crystalline phase at 2θ values of 25.77°, 37.72°, 47.92°, 53.9°, 55.02°, 62.57°, 68.4°, 70.3°, and 74.9° are corresponding to the (101), (004), (200), (105), (211), (204), (116), (220) and (215)^29^. The XRD results of TiO_2_ doped by Co and C nanocomposites demonstrate the lack of any free cobalt phases, which implies that all the cobalt is a fuse in anatase crystallites. XRD results show that there is a lack of obvious carbon peaks, indicating that the carbon in the composite has a low degree of crystallinity. Surprisingly, the two samples’ Co-TiO_2_ and Co-TiO_2_/C diffraction patterns resembled those of virgin TiO_2_, with a minor change in the position of the peak. The slight shift in peak position in the XRD pattern is the result of the modification in structure at the local level around Ti^4+^ resulting from the substitution of Co^3+^ and Co^2+^. This suggests that Cobalt dissolved within the lattice. Another notable change with the inclusion of cobalt is the change in the color of TiO_2_. All of the XRD patterns revealing that the average size of the nanocrystalline particles of pure TiO_2_ changed when cobalt or cobalt/carbon is added. According to Scherrer's equation (Fig. 3)^29^, the average crystallite size of prepared samples: pure TiO_2_ (TiO_2_ nanoparticles), single doped TiO_2_ (Co-TiO_2_ nanocomposite), and double doped TiO_2_ (Co-TiO_2_/C nanocomposite) were 11.7, 12.4, and 13.24 nm, respectively. The samples' increased crystal size emphasizes that cobalt is replacing Titanium in the lattice of TiO_2_ (anatase phase). An examination of the main peak in XRD diffraction (101) in Fig. 2 (inset) when magnified, shows a significant shift towards higher 1 theta values when cobalt and carbon are introduced as dopants. Additionally, the average crystallite size of double-doped TiO_2_ by C and Co (Co-TiO_2_/C) material is found to be greater in comparison to that of the single-doped TiO_2_ by Co only (Co-TiO_2_). These findings suggest that there is a synergistic effect between carbon and cobalt when they are combined together. The XRD results reveal that the average crystallite size of the two manufactured materials doped by (Co) and (Co, C) are almost identical. Furthermore, their positions of diffraction peaks are similar, which implies that carbon doping has a minor impact on the crystal structure^30,31^.

#### Raman shifts analysis

**Figure 4:**
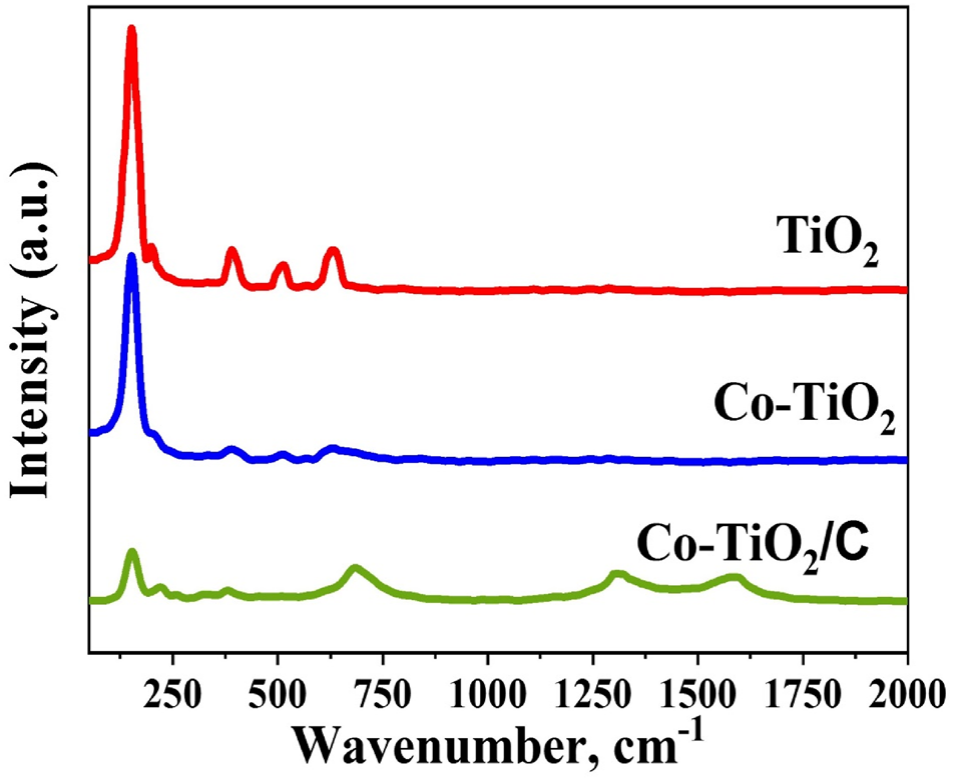
Raman spectrum of the fabricated nanocomposites samples.

Figure 4 shows the Raman spectrum of three fabricated samples in the 50–2000 cm^−1^ range. The Peaks which apperant at 151.5, 391.5, 513, and 631.5 cm^−1^ were assigned to TiO_2_ (anatase crystalline phase) modes *Eg*, *B1g*, *A1g*, and *Eg*, respectively^32^. All peaks show a significant decrease in intensity after cobalt doping, along with some shift in peak position. Although these findings imply that the anatase phase was not entirely altered by the Co, they also reveal a significant long-order crystallite distortion, which agrees with the XRD findings. The substitution of Ti^4+^ by Co^3+^ and the disparity in their cationic charges could explain this distortion. Furthermore, doping by Co and C also correlated to the formation of vacancies in the oxygen chain, which significantly affects the vibration of the bond between Ti and O (Ti–O). It is substantial to point out while the size of the nanoscale crystalline phase, which influences the frequency of shifting brought on by phonon confinement, limits the peak position shift. The *A1g* peak shows a clear shift in location and an obvious rise in intensity. Modification in the *A1g* peak indicates the formation of more vacancies in the oxygen chain, mainly likely as a consequence of the reaction with carbon in the hydrothermal reaction. According to the strong Raman peak at 689 cm^−1^, the anatase Eg modes shifted as a result of being doped by carbon and cobalt^33^. Even after the calcination of the material containing carbon in the air, the existence of carbon can be established by seeing peaks at 1300 cm^−1^ and 1850 cm^−1^ which refer to D and G bands, respectively. Given that the ratio of the intensity of two peaks D and G (I_D_/I_G_) = 1.3, the carbon is most likely amorphous in form^34^.

#### SEM and TEM analysis

**Figure 5:**
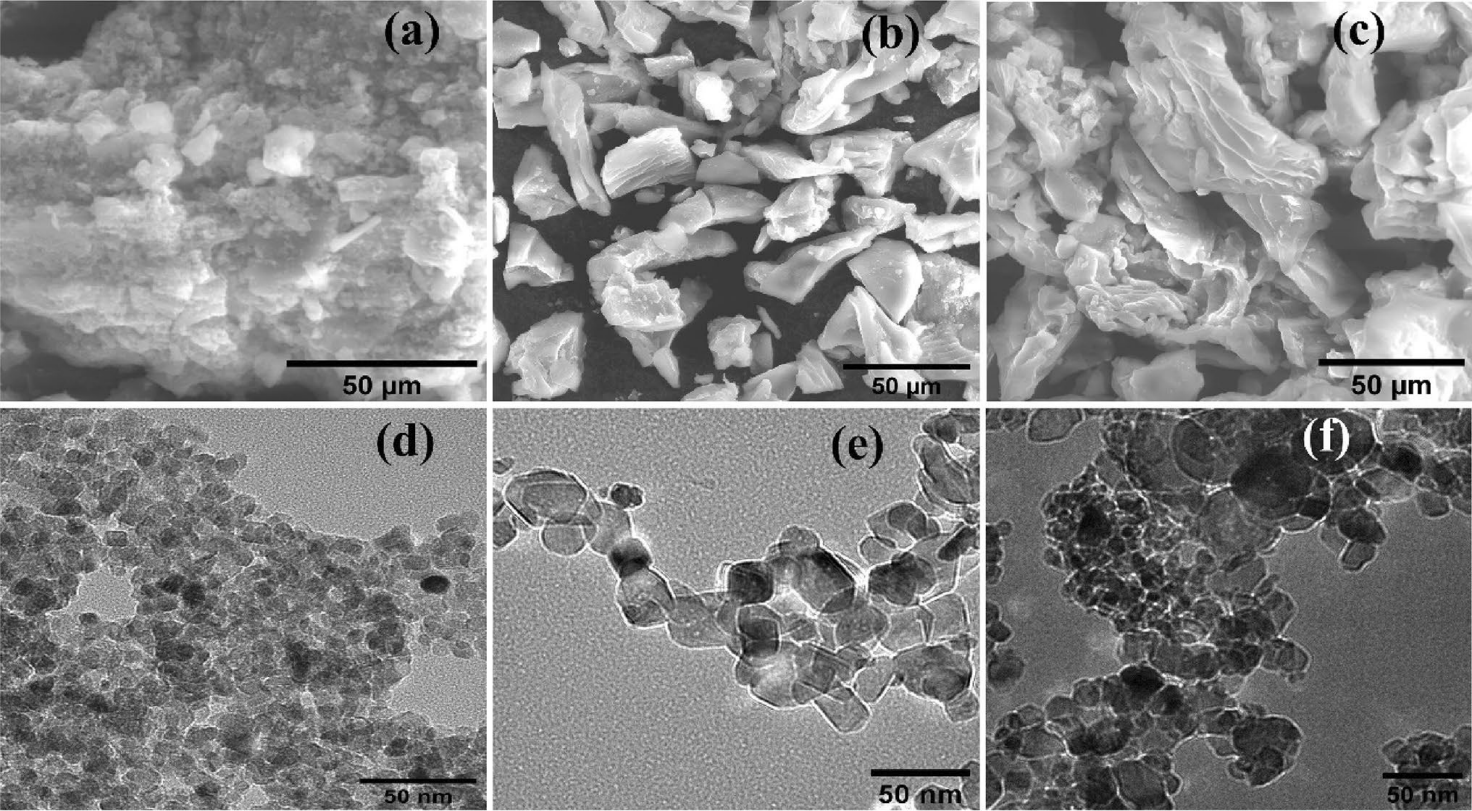
(**a**–**c**) SEM image for TiO_2_ nanoparticles, single doped TiO_2_ (Co-TiO_2_), and double doped TiO_2_ (Co-TiO_2_/C), respectively. (**d**–**f**) TEM image for TiO_2_ nanoparticles, single doped TiO_2_ (Co-TiO_2_), and double doped TiO_2_ (Co-TiO_2_/C), respectively.

**Figure 6:**
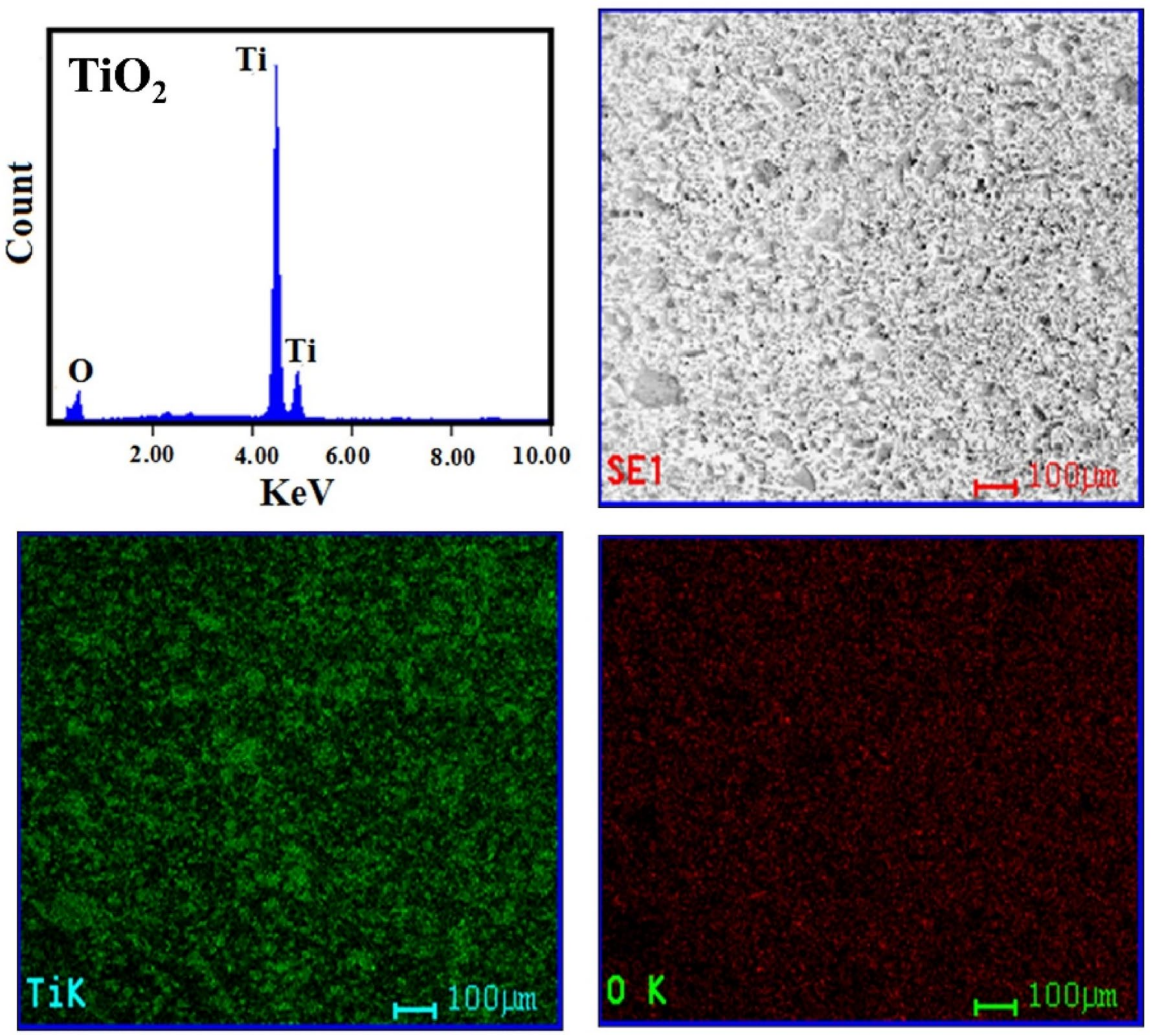
Elemental analysis and mapping (EDX) of the fabricated TiO_2_ nanoparticles.

**Figure 7:**
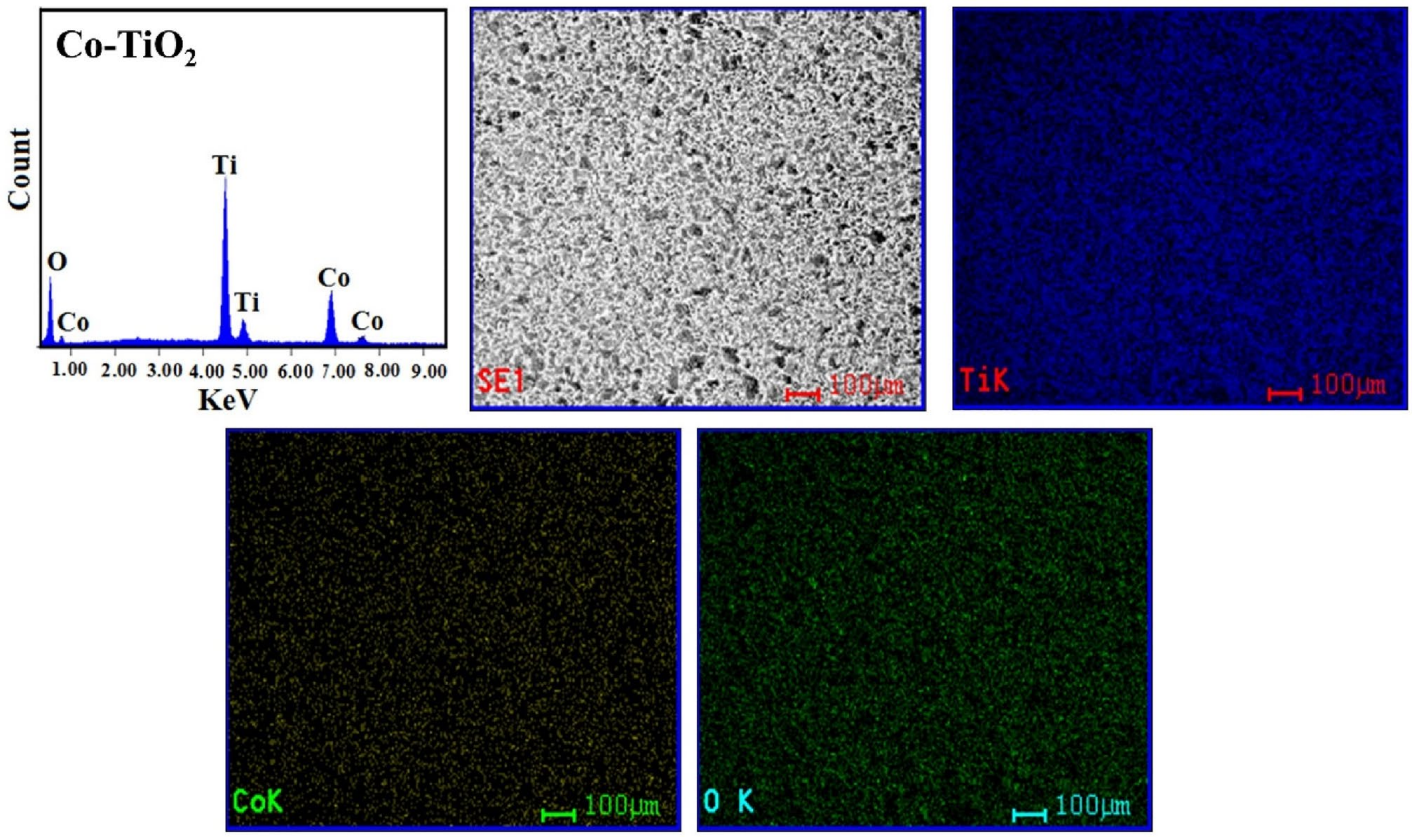
Elemental analysis and mapping (EDX) of the fabricated Co-TiO_2_ nanocomposite.

**Figure 8:**
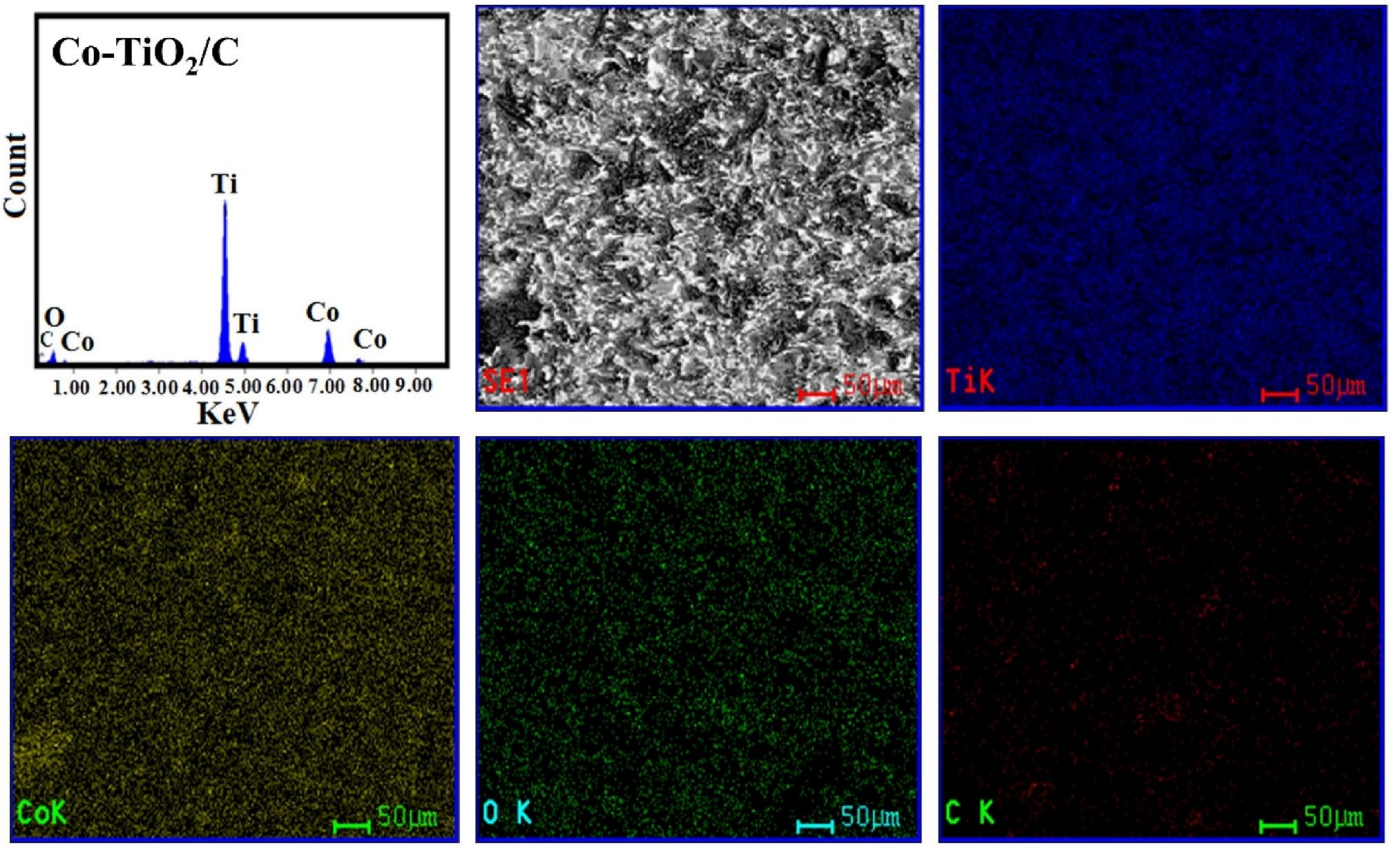
Elemental analysis and mapping (EDX) of the fabricated Co-TiO_2_/C nanocomposite**.**

Figure 5a–c shows SEM of TiO_2_ nanoparticles, TiO_2_ doped by Co (Co-TiO_2_), and when adding carbon (Co-TiO_2_/C) after 500 °C calcination. The results show that nanoparticle clusters form in all samples. Surprisingly, the two samples of TiO_2_ which were doped by (Co) and (Co, C) form 2D sheets. Undoped TiO_2_ nanoparticles, on the other hand, tend to form more irregular shapes when clustered. After the addition of cobalt and carbon, the appearance of distinct nanosheets indicates a predilection for development in particular directions. After the addition of cobalt, there is most likely a rise in state density perpendicular to the C-axes; in addition, partial oxygen removal occurs due to the presence of carbon. The 2D sheets are only weakly attached to one another to create a zigzag pattern with pores, which is seen in Fig. 5b,c. The TEM images (Fig. 5d–f) provide additional information concerning fabricated materials' morphology. According to the findings, each of the three types of material put to the test is made up of nanoparticles that have parallel sides and measure approximately 23 nm in size on average. At the edges, the particles start fusing together after adding Co which forms 2D nanolayer sheets. Then, when glucose has been added as a carbon source, the clusters and average particle size were increased without any distortion in the anatase phase. The EDX spectra and element mapping (Fig. 6) show pure TiO_2_, Fig. 7 indicates the existence of (Co) element in the single doped prepared sample (Co-TiO_2_), and Fig. 8 indicates the existence of (Co, C) elements in the double doped prepared sample (Co-TiO_2_/C). There were no peaks associated with Co or C in the XRD patterns. As a result, the Co ions in the single-doped case (Co-TiO_2_) and (Co, C) ions in the double-doped case (Co-TiO_2_/C) are distributed among the titanium dioxide crystallites (anatase phase) in a uniform form. In Table 1, the elemental analysis composition of TiO_2_ nanoparticles after doping by Co and C can be determined.

**Table 1:** Element analysis of TiO_2_ nanoparticles, single doped TiO_2_ (Co-TiO_2_) nanocomposite, and double doped TiO_2_ (Co-TiO_2_/C) nanocomposite.

Element	wt%		
	TiO_2_	Co-TiO_2_	Co-TiO_2_/C
Ti	61.9	56.1	55.4
O	38.1	40.2	36.2
Co	–	3.7	3.6
C	–	–	4.8

### Radiation shielding properties

**Figure 9:**
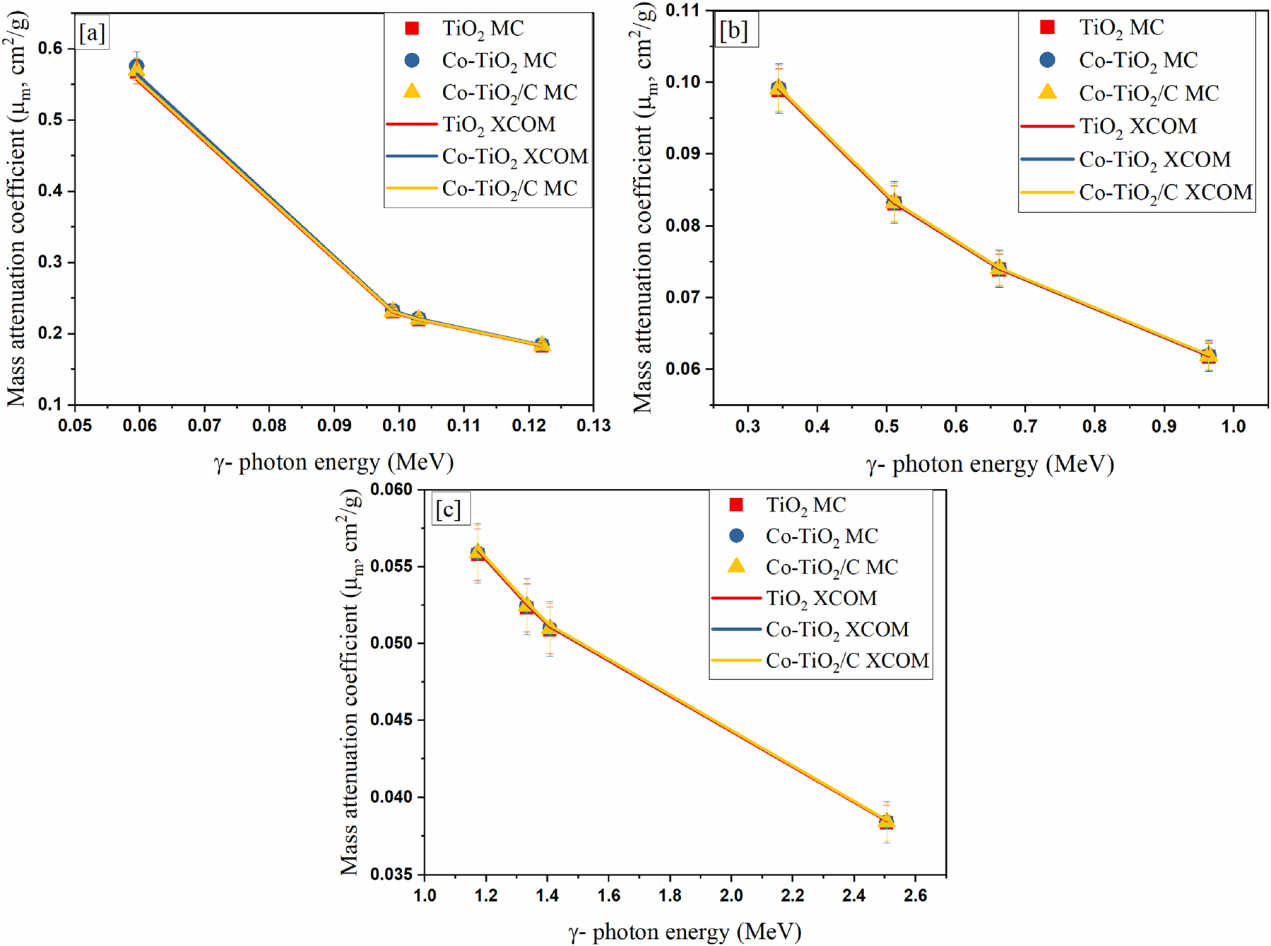
The mass attenuation coefficient for the fabricated composites.

The µ_m_ and µ are the main important parameters that describe the capacity of newly synthesized cobalt-doped titania nanosheets on resisting the transport of photons. Both µ_m_ and µ were evaluated for the fabricated nanocomposites (pure TiO_2_ nanoparticles, single doped TiO_2_ (Co-TiO_2_) nanocomposite, and douple doped TiO_2_ (Co-TiO_2_/C) nanocomposite) using the MCNP code as well as the XCOM software program over a wide γ-ray spectrum (E_γ_, MeV) ranging from 0.059 to 2.506 MeV. The µ_m_ behavior is depicted in Fig. 9 at various γ-ray interactions regions [a] photoelectric interaction, [b] Compton scattering, and [c] pair production interactions. According to Fig. 9a, the µ_m_ values decreased by 68% for both manufactured composites (pure TiO_2_ nanoparticles, single doped TiO_2_ (Co-TiO_2_) nanocomposite, and douple doped TiO_2_ (Co-TiO_2_/C) nanocomposite) with increasing E_γ_ values. The simulated µ_m_ values decreased between 0.567 and 0.183 cm^2^/g for the TiO_2_ NPs, between 0.576–0.184 cm^2^/g for the Co-TiO_2_ composite, and between 0.569 and 0.183 cm^2^/g for the Co-TiO_2_/C composite. The photoelectric cross section varied inversely with the third power of E_γ_, explaining the large decrease in the µ_m_ values. Figure 9b shows a decrease in the µ_m_ values between 0.0989–0.0618 cm^2^/g for the TiO_2_ NPs, between 0.0991 and 0.0619 cm^2^/g for the Co-TiO_2_ composite, and between 0.0991 and 0.0619 cm^2^/g for Co-TiO_2_/C with raising the E_γ_ values between 0.344 and 0.964 MeV. The presented reduction behavior was studied using the cross-section of the Compton scattering, which varies inversely with E_γ_. Figure 9c depicts a slight reduce in the µ_m_ values associated with any increase in the E_γ_ values within the energy interval 1.173–2.506 MeV. The illustrated slight reduction is due to the fact that the CS interaction is still the predominant interaction also between 1.173 and 2.506 MeV. Therefore, the PE, and CS interactions only appeared during the result analyses while the pair production interaction (PP) was not observed in the studied energy range. The delayed appearance of PP interaction in the current work is attributed to the relatively small density of the manufactured composites. The PP interaction in the current work starts at gamma-ray energy higher than 3 MeV where the high variation in the gamma energy (in the PP interval) causes a very slight increase in the µ_m_ values. This behavior is attributed to the proportionality of the PP cross-section with Log E.

The simulated results using the MCNP were confirmed using the theoretical free program XCOM as illustrated in Fig. 9. The obtained results showed an agreement with the simulated data with a range between ± 2%.

**Figure 10:**
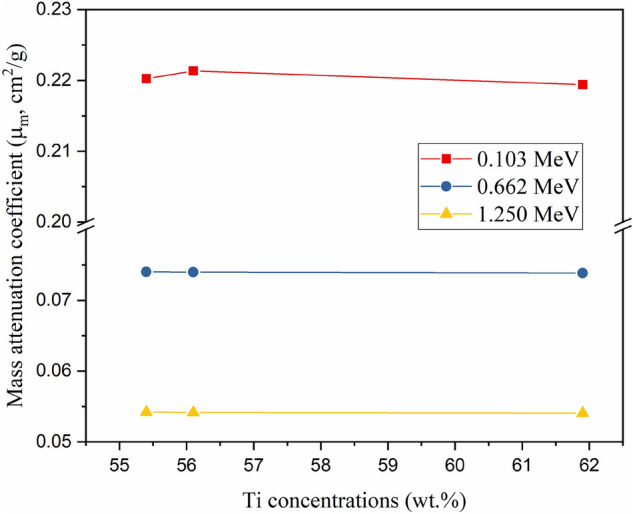
Variation of the mass attenuation coefficient (µ_m_, cm^2^/g) versus the Ti concentrations at 0.103, 0.662, and 1.250 MeV.

Also, the modification of the titanium dioxide (TiO_2_ NPs) with Co and C nanoparticles has a slightly enhancing effect on the µ_m_ values. Figure 10 describes the variation of the µ_m_ values versus the Ti nanoparticle concentrations in the fabricated composites. In the low energy interval (i.e., E_γ_ of 0.103 MeV) the µ_m_ values increased slightly with decreasing Ti nanoparticles in the fabricated composites while in the high photon energies, the µ_m_ values increased with raising the Ti nanoparticle. This behavior is attributed to the Co nanoparticles where the decrease in the Ti nanoparticles concentrations was accompanied by an increase in the Co nanoparticles concentration. Hence, the Co nanoparticles have a µ_m_ value higher than that recorded for the Ti nanoparticles at low E_γ_ values while the µ_m_ values for the Co nanoparticles are comparable to the µ_m_ values of Ti at high E_γ_ values. For instance, the µ_m_ for Co is 1.343cm^2^/g at a low E_γ_ of 0.059 MeV while it is 0.782cm^2^/g for Ti element at the same E_γ_ value. Moreover, the Co's µ_m_ value is 0.496 cm^2^/g at E_γ_ of 1.408 MeV, which is comparable to the Ti's µ_m_ value of 0.495 cm^2^/g^35^. Then decreasing the concentration of Ti nanoparticles in the manufactured nanocomposites was associated with an increase in the µ_m_ values at low γ-photon energies while the mentioned trend was reversed for the high γ-photon energy. Because of the low concentrations of Co and C nanoparticles added to the TiO_2_ nanoparticles, the variation in simulated µ_m_ values is relatively small.

**Figure 11:**
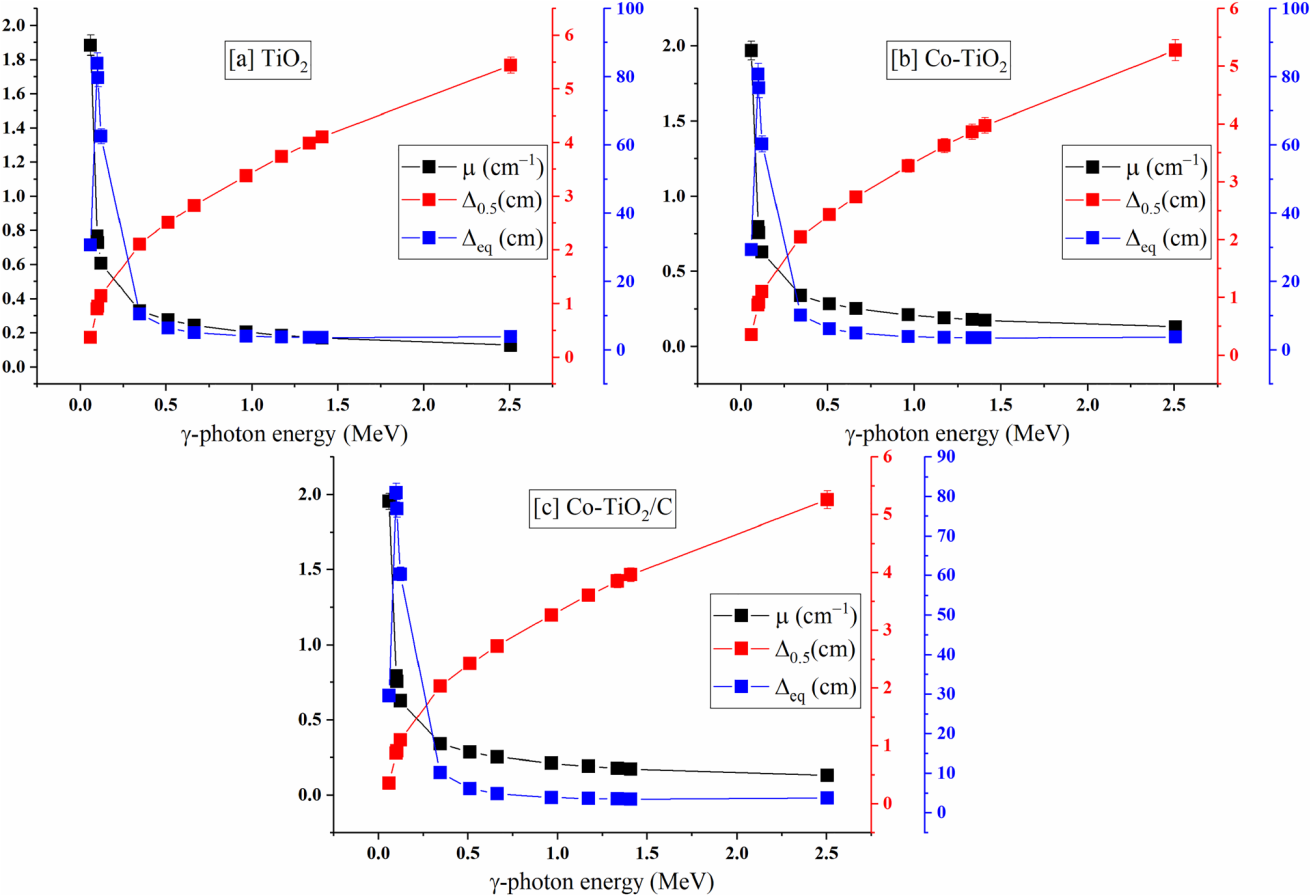
The linear attenuation coefficient, half-value thickness, and lead equivalent thickness for the prepared composites.

The Δ_eq_ describes the synthesized composites’ thickness which is able to attenuate the same photon number as a 1 cm thickness of the pure lead (Pb). Figure 11 depicts the change in the µ, Δ_0.5_, and Δ_eq_ values versus E_γ_ values for the fabricated composites [a] TiO_2_ nanoparticles, [b] single doped TiO_2_ (Co-TiO_2_), and [c] double doped TiO_2_ (Co-TiO_2_/C) versus. Figures 11 a, b, and c show a decrease in µ and Δ_eq_ values and increase the Δ_0.5_ values. The evaluated µ values decreased shapely between 1.884 and 0.127 cm^−1^ (for TiO_2_ NPs), between 1.970–0.131 cm^−1^ (For Co-TiO_2_/C nanocomposite), and between 1.955 and 0.132 cm^−1^ (for Co-TiO_2_/C nanocomposite) with raising the E_γ_. The reduction of the µ was caused by the effect of PE, CS, and PP interactions. The highest calculated Δ_eq_ values are 83.978, 80.772, and 80.977 cm for the fabricated pure TiO_2_ nanoparticles, single doped TiO_2_ Co-TiO_2_ composite, and double doped TiO_2_ Co-TiO_2_/C composite, respectively. The calculated Δ_eq_ decrease rapidly as the E_γ_ values increase due to the large decrease in the Pb’s µ values compared to the decrease achieved in the fabricated composites’ µ value. Between 0.059 and 0.122 MeV, the Δ_eq_ increased by a factor of ≈ 103% for the fabricated composites. The mentioned increase in the Δ_eq_ is attributed to the low drop in Pb’s µ compared to the reported drop in the fabricated composites µ values. The Pb’s µ droped by a factor of ≈ 34%, whereas the tested composites’ µ values decreased by a factor of ≈ 67% when the E_γ_ was raised between 0.059 and 0.122 MeV. After that, in the intermediate energy interval between 0.344 and 0.964 MeV, the Δ_eq_ values were reduced by factors of ≈ 62%. This reduction is due to a large reduction in Pb’s µ values compared to the moderate µ values reduction for the fabricated composites. In the CS interaction interval, the Pb’s µ value droped by 76% and the µ for the synthesized composites decreased by 37.57, 37.60, and 37.57% for TiO_2_ nanoparticles, single doped TiO_2_ (Co-TiO_2_), and double doped TiO_2_ (Co-TiO_2_/C). Because the µ values for Pb and the fabricated composites are independent of the γ-photon energy, there is no significant variation in the Δ_eq_ values with variation in the E_γ_ values in the high E_γ_ interval between 1.173–2.506 MeV. The Δ_0.5_ values show an increase with raising the E_γ_ values due to the reduction recorded for the µ values, where the Δ_0.5_ values are inversely proportional to the µ (see Eq. 3).

**Figure 12:**
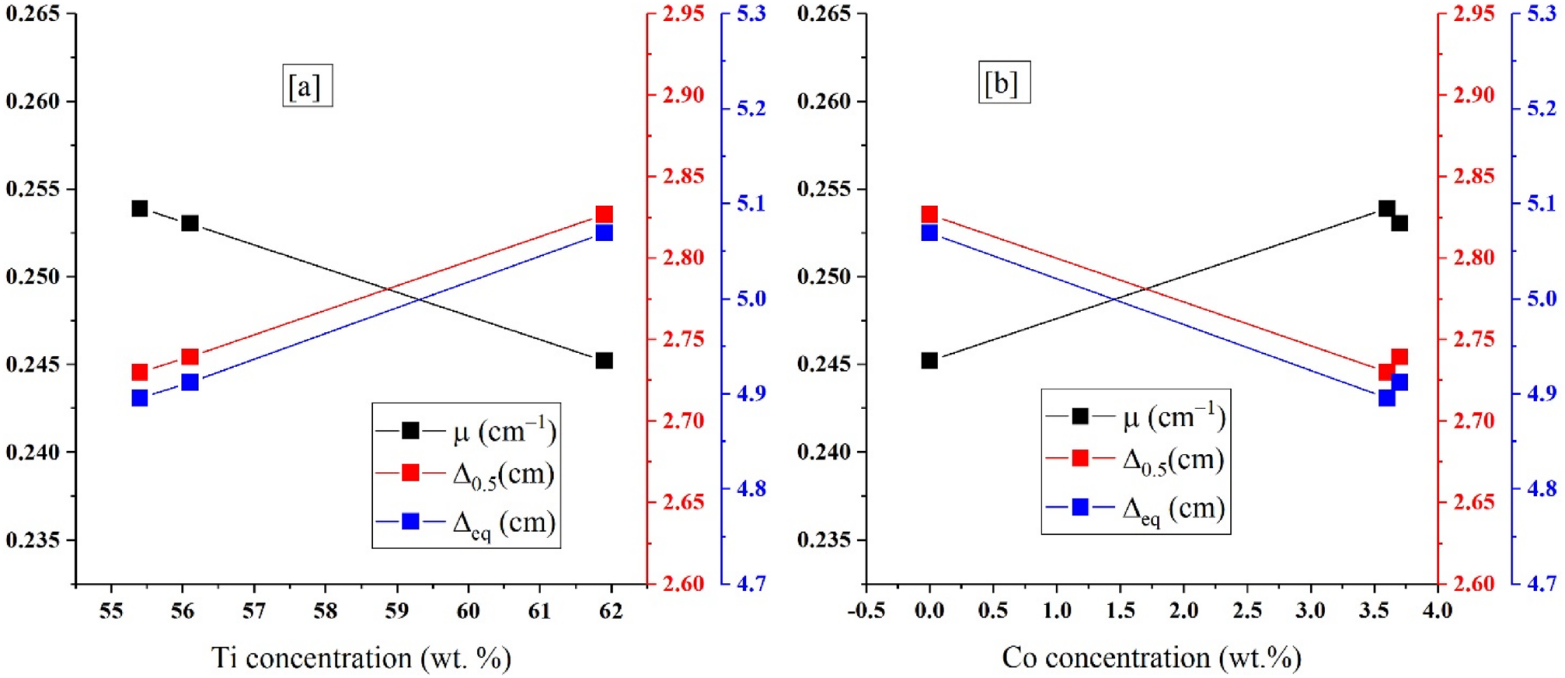
Variation of the linear attenuation coefficient, half-value thickness, and lead equivalent thickness versus Ti and Co concentrations.

The change in µ, Δ_0.5_, and Δ_eq_ values vs. the amounts of Ti^4+^ and Co^3+^ nanoparticles at E_γ_ of 0.662 MeV was given in Fig. 12. The µ values decreased with raising the Ti concentrations while they increased with raising the Co concentration. While, the Δ_0.5_, and Δ_eq_ increased with raising the Ti concentrations while they decreased with raising the Co concentrations at E_γ_ of 0.662 MeV. The Δ_0.5_ values increased slightly from 2.730 to 2.827 cm with raising the Ti^4+^ concentration from 55.4 to 61.9 wt% while it decreased with raising the Co concentration from 0 to 3.7%. Furthermore, the Δ_eq_ values at the same E_γ_ of 0.662 increase from 4.896 to 5.069 cm with raising the Ti nanoparticle concentration from 55.4 to 61.9 wt% while it decreases from 5.069 to 4.896 with raising the Co^3+^ nanoparticle concentration from 0 to 3.7 wt%. The illustrated behaviors for µ, Δ_0.5_, and Δ_eq_ were attributed to the partial replacement of Ti^4+^ nanoparticles with Co^3+^ nanoparticles where the partial replacement of the Ti by Co nanoparticles causes an increase in the electron density of the composites which creates more resistance for passing the incident photons.

**Figure 13:**
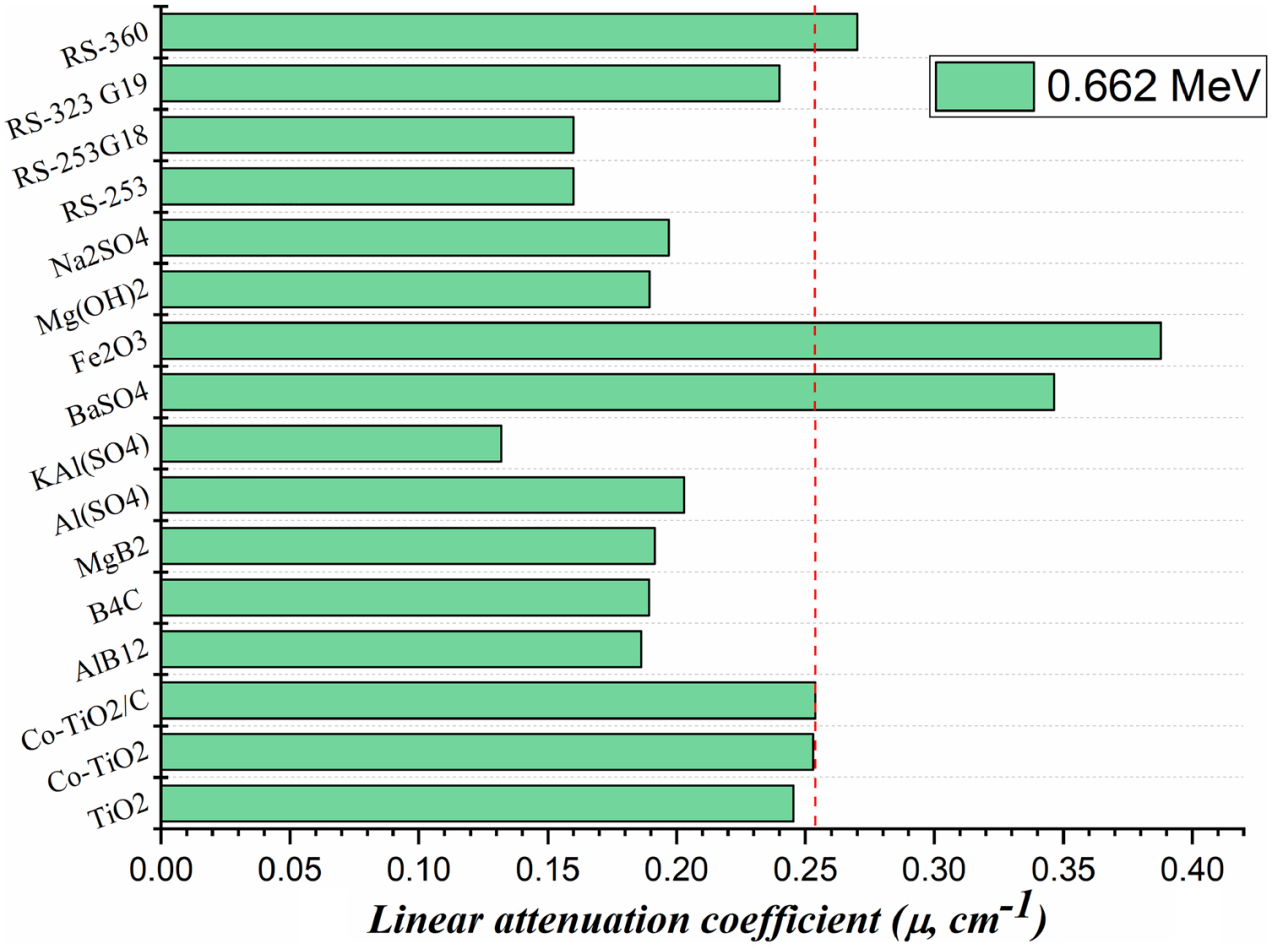
Comparison of the linear attenuation coefficient for the fabricated composites to the linear attenuation of previously reported compounds and commercial shielding glasses.

Figure 13 illustrates a comparison between the fabricated composites µ values and the µ values for some previously reported composites and common commercial shielding glasses at E_γ_ of 0.662 MeV. The fabricated composites TiO_2_, Co-TiO_2_ and Co-TiO_2_/C have µ values of 0.245 cm^−1^, 0.253 cm^−1^, and 0.254 cm^−1^. These values are higher than reported for AlB_12_ (0.186 cm^−1^), B_4_C (0.189 cm^−1^), MgB_2_ (0.192 cm^−1^), Al (SO_4_) (0.203 cm^−1^), KAl (SO_4_) (0.132 cm^−1^), Mg(OH)_2_ (190 cm^−1^), and Na_2_SO_4_ (0.197 cm^−1^) compounds^36,37^. Additionally, the fabricated composites µ values are higher than the common commercial glasses’ RS-253, RS-253 G18, and RS-323 G19 with µ values of 0.16 cm^−1^, 0.16 cm^−1^, and 0.24 cm^−1^, respectively^38^. In contrast, the fabricated compounds have µ values less than that reported for BaSO_4_, Fe_2_O_3_^36^, and RS-360^38^ with µ values of 0.347 cm^−1^, 0.388 cm^−1^, and 0.27 cm^−1^, respectively. The high amount of heavy metals Ba, Fe, and Pb is responsible for the high linear values for Ba_2_SO_4_, Fe_2_O_3_, and RS-360, with the last containing almost 45 wt% PbO.

**Figure 14:**
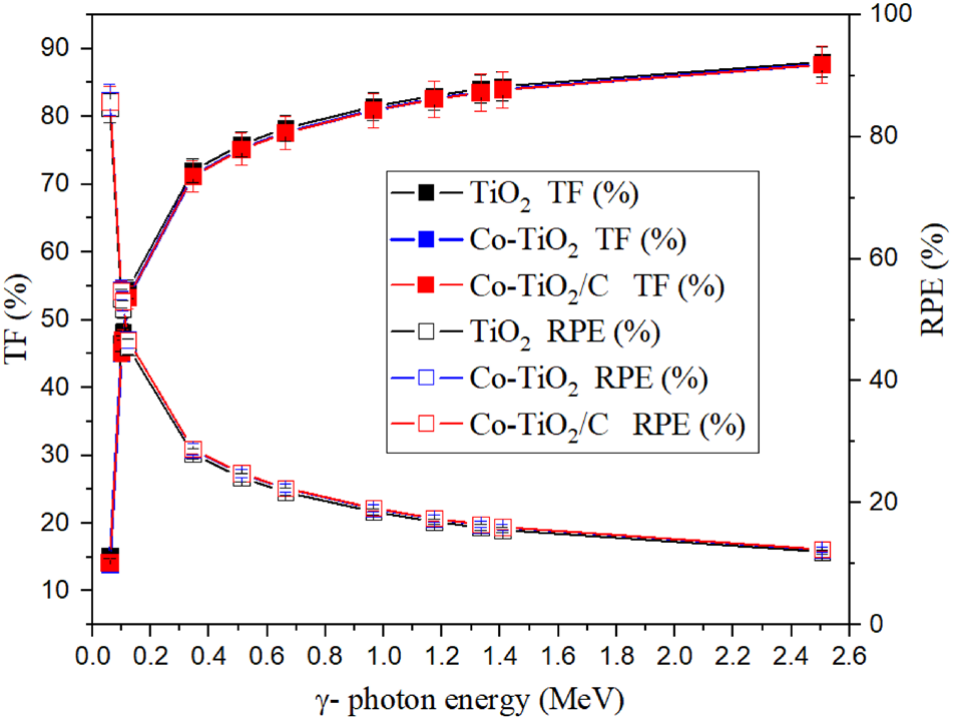
Dependence of the transmission factor and the radiation protection efficiency on the γ-photon energy for the fabricated composites.

The TF and RPE for the selected composites were exhibited in Fig. 14. A decrease in the TF values between 15.2 and 88.0% (for composite TiO_2_), between 13.9 and 87.7% (for Co-TiO_2_ composite), and between 14.2 and 87.8% (for Co-TiO_2_/C composite) when the E_γ_ increased from 0.059 to 2.506 MeV. On the other hand, the RPE decreases from 84.8 to 12.0% (for composite TiO_2_), from 86.1 to 12.3% (for composite Co-TiO_2_), and from 85.8 to 12.3% (for composite Co-TiO_2_/C) when the E_γ_ varied between 0.059 and 2.506 MeV. The previously discussed behaviors for both parameters are attributed to the penetration ability of the incident γ-photons, where raising the energy causes an increase in the penetration power of the photons due to the decrease of the photon wavelength. Therefore, the emitted photons (N_o_) pass the tested composites with a low number of collisions with the surrounding electrons. Thus, the N_t_ increase is associated with a decrease in the number of photons absorbed in the fabricated composites (N_a_). The mentioned increase in the N_t_ as well as the decrease in the N_a_ photons give rise to the increase in the TF and decrease in the RPE with raising the γ-photon energies.

**Figure 15:**
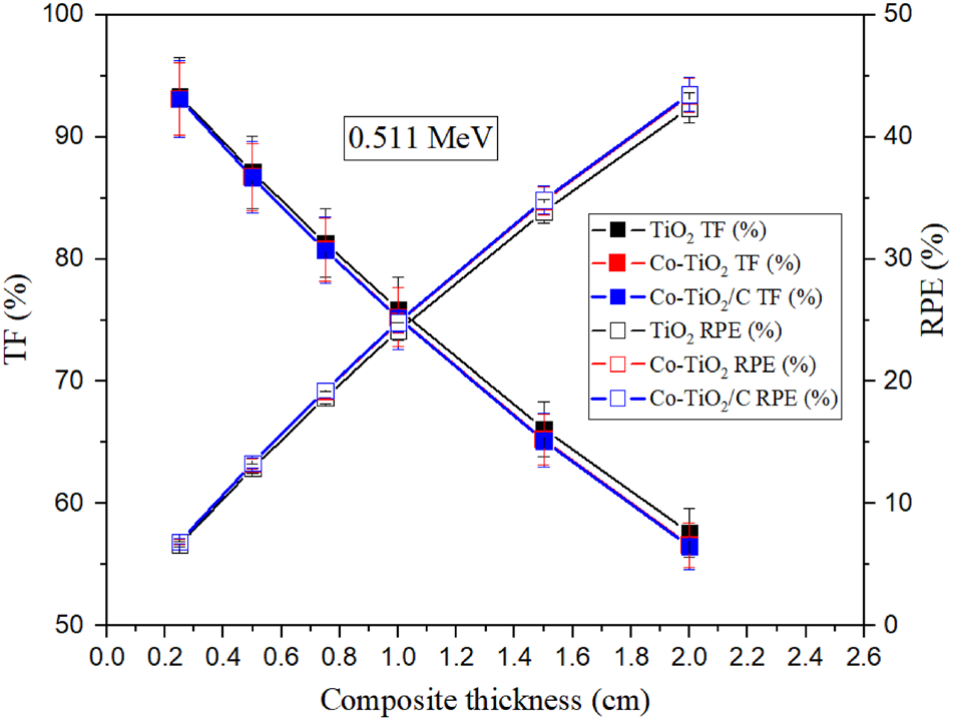
Dependence of the transmission factor and the radiation protection efficiency on the composite thickness.

The dependence of TF and RPE values on the composites’ thickness was illustrated in Fig. 15. The TF values decreased and the RPE increases for all fabricated composites when the composite thickness grew between 0.025 and 2 cm. When the thickness ranged between 0.25 and 2 cm, the TF values decreased linearly from 93.3 to 57.6% (for TiO_2_ composite), between 86.7 and 56.6% (for Co-TiO_2_ composite), and between 86.7 to 56.5% (for Co-TiO_2_/C composite). But, the RPE increased linearly from 6.7 to 42.4% (for TiO_2_ composite), from 6.9 and 43.4% (for Co-TiO_2_ composite), and from 6.9 to 43.5% (for Co-TiO_2_/C composite) when the thickness of the composite raised from 0.25 to 2 cm at a gamma photon energy of 0.511 MeV. Raising the composite thickness causes an increase in the path length of incident γ-photons inside the fabricated composites. Therefore, the possibility of the incident photons interacting with the surrounding electrons increases. Thus, the consumed energy by incident photons increased inside the material with higher thicknesses than the lower one, resulting in a significant increase in RPE values accompanied by a corresponding decrease in TF values^39^.

## Conclusions

A Cobalt-doped TiO_2_ nanocomposite with more vacancies in the oxygen chain was synthesized based on a hydrothermal method in one step and the formation and crystallinity of the synthesized composite were confirmed using XRD in 2D sheets with an average crystal size of less than 13 nm. Moreover, the µ_m_ of the cobalt-doped titania nanocomposites was estimated using MCNP5 between 0.059 and 2.506 MeV. The simulated values of the mass attenuation coefficient were confirmed using the XCOM program, where the difference between the XCOM and MCNP results ranged between ≈ ± 2%. The mass attenuation coefficient decreased from 0.567 to 0.038 cm^2^/g (TiO_2_ composite), 0.567 to 0.038 cm^2^/g (Co-TiO_2_ composite), and from 0.570 to 0.038 cm^2^/g (Co-TiO_2_/C composite) in the studied energy region. The mass attenuation coefficient values increase with decreasing the Ti^4+^ ions in the fabricated composites at low γ-photo energy while it has no considerable changes with substitution of Ti^4+^ by Co^3+^ ions at intermediate and high γ-photon energy. Furthermore, the µ is slightly increased by raising the Co^3+^ ions between 0 and 3.7 wt% in the fabricated composites. The mentioned increase in the µ values was associated with a slight decrease in Δ_0.5_ and Δ_eq_ values. Based on the concluded results, the fabricated composites-based TiO_2_ nanocomposites can be used as fillers to improve the gamma-ray shielding capacity of polymers, ceramics, and paint materials. The fabricated composites have a good shielding capacity in low and intermediate gamma-ray energy intervals but it is not suitable for high gamma-ray energy applications.

## Data Availability

The data that support the findings of this study are available from the corresponding author upon reasonable request.
